# Performance analysis of linearization schemes for modelling multi-phase flow in porous media

**DOI:** 10.1038/s41598-024-66628-3

**Published:** 2024-07-07

**Authors:** Abdul Salam Abd, Ali Asif, Ahmad Abushaikha

**Affiliations:** Division of Sustainable Development, College of Science and Engineering, Education City, Hamad Bin Khalifa University, Qatar Foundation, P. O. Box 5825, Doha, Qatar

**Keywords:** Linearization, Nonlinear solvers, Reservoir simulation, Numerical analysis, Computational science, Fluid dynamics

## Abstract

Reservoir simulation is crucial for understanding the flow response in underground reservoirs, and it significantly helps reduce uncertainties in geological characterization and optimize methodologies for field development strategies. However, providing efficient and accurate solutions for the strong heterogeneity remains challenging, as most of the discretization methods cannot handle this complexity. In this work, we perform a comprehensive assessment of various numerical linearization techniques employed in reservoir simulation, particularly focusing on the performance of the nonlinear solver for problem dealing with fluid flow in porous media. The primary linearization methods examined are finite difference central (FDC), finite forward difference (FDF), and operator-based linearization (OBL). These methods are rigorously analyzed and compared in terms of their accuracy, computational efficiency, and adaptability to changing reservoir conditions. The results demonstrate that each method has distinct strengths and limitations. The FDC method is more accurate particularly in complex simulations where strong heterogeneity are introduced but is generally slower in convergence. The OBL on the other hand, is more efficient and converges quickly, which makes it suitable for scenarios with limited computational resources and simple physics, while the FDF method provides a balanced combination of precision and computational speed, contingent upon careful step size management of the derivative estimations. This paper aims to guide the selection of appropriate linearization techniques for enhancing nonlinear solvers’ accuracy and efficiency in reservoir simulation .

## Introduction

A petroleum reservoir is an underground structure containing hydrocarbons, which can be confined either by its physical shape or by its stratification. It is complicated to predict how fluid moves in this environment as opposed to standard pipes or channels. This complexity arises because, in contrast to the set paths in pipes, flow directions in these porous structures are unpredictable, complicating the task of estimating the flux potential based on pressure^1^. This phenomena of flow in porous media is complex and occurs in various applications as well such as groundwater flow and oil production from subsurface reservoir. This means that understanding and analyzing the behavior of fluid flow in porous media is crucial for making informed decisions and optimizing processes in these fields.

In the process of reservoir simulation, once the governing equations are discretized over space and time, the next step is to simplify their nonlinearity through linearization^2^. The equations used in reservoir simulation are inherently nonlinear^3^. In order to create discrete approximations that incorporate a degree of implicitness, it becomes necessary to solve simultaneous systems of nonlinear equations. The methods for solving these nonlinear equations involve a process of linearization, which simplifies the equations into a linear form. Subsequently, these resulting linear systems are solved to find the primary variables in the governing equation of interest. This approach is a fundamental aspect of reservoir simulation, allowing for the effective handling of the complex and nonlinear nature of reservoir behaviors and conditions^4^.

The implementation of the formulation and linearization component in reservoir simulation software is often considered the least flexible, most labor-intensive, and most prone to errors^5–8^. This crucial aspect of the software involves computing the residual, denoted as, *r*, and the Jacobian matrix, *J*, for a given set of unknown variables. These computations are integral parts of the code that demand extensive expertise in both the physical models of reservoir simulation and their discrete approximations. The divide between manual code optimization and the clarity of code significantly complicates the task of developing reservoir simulators for new and evolving computer hardware. Programmers tasked with developing the physical models of the software generally focus more on the accuracy and correctness of the implementation rather than optimizing for performance. Their expertise lies more in the domain of understanding and applying the complex physical models rather than in fine-tuning the code for enhanced computational efficiency. This can lead to challenges in ensuring both the reliability and the performance of the simulation software.

In reservoir simulation, the complexity of the physics involved dictates the minimum number of independent variables and equations required to define the thermodynamic state of the system. For instance, in isothermal compositional systems, the thermodynamic state is determined by a number of independent variables equal to the number of components in the system^9^. All other properties can then be derived using these independent variables along with constitutive relations or algebraic constraints. However, a challenge arises because these algebraic constraints are often implicit nonlinear functions, meaning they cannot be directly solved without further manipulation. Specifically, solving for one variable in terms of others often requires computing the inverse of these nonlinear functions, which can be computationally intensive and complex. A common approach to handle this is by integrating these constraints with the fundamental set of governing equations, forming a system known as partial differential algebraic equations. The process of selecting independent variables, secondary variables, algebraic constraints, and aligning unknowns with equations is known as formulating a problem. Different formulations might exhibit distinct numerical properties, but ideally, they all converge to the same correct solution^10^. This leads to a significant interest in examining the characteristics of various formulations as certain problems can be better addressed by specific formulations^11–13^.

Modern simulation software must be versatile enough to accommodate diverse interests and approaches. This means that such software should have the capability to implement different formulations, allowing users to choose the one most suited to their specific problem. Once a specific formulation and its corresponding principles are selected in reservoir simulation, there are three distinct methods to implement routines for evaluating the Jacobian matrix. One such method is analytical differentiation, which involves the explicit calculation and coding of all derivatives and the assembly of the Jacobian matrix. This method is commonly used in most commercially and publicly available simulators^6,7,14^. The process of analytical differentiation, while thorough, is known to be quite laborious and prone to errors. However, it yields efficiently computed Jacobian matrices that are exact up to the limitations of rounding errors. Despite this precision, the primary disadvantage of this approach lies in its inflexibility. Any modifications to the numerical formulations or physical models can necessitate extensive rewrites of significant portions of the software. These rewrites might span across various modules, from property calculations to the formulation of equations, and even the setup of solvers.

The second commonly used approach is Automatic Differentiation (AD) that enhances computer programs by automatically calculating derivatives. It works by analyzing the parse-tree of expressions in the program and applying a set of simple rules to compute derivatives^15^. AD is known for its flexibility and general applicability, and it can achieve accuracy that is limited only by the precision of the machine it is running on. Despite these advantages, the efficiency of AD can vary depending on the specific problem it is applied to as it often requires problem-specific adjustments and fine-tuning. The AD-based library (ADETL) was introduced in reservoir simulation by Younis et al.^16^, leading to the creation of the Automatic Differentiation General Purpose Research Simulator (ADGPRS)^17,18^. Subsequently, the AD approach gained traction in research-oriented reservoir simulation frameworks^19^. While the AD method is beneficial for its flexibility, it inherently includes computational overhead, impacting the efficiency of reservoir simulations^20^.

Numerical differentiation is another approach used to approximate the Jacobian matrix in computational simulations, including those in reservoir engineering. This technique typically employs truncated series expansions, often using divided differences, and can incorporate methods like coloring and separation algorithms based on sparsity graphs^21^. The use of numerical derivatives generally offers versatility in nonlinear formulations yet simulations relying on these derivatives might compromise robustness and efficiency^22^. Despite its flexibility, numerical differentiation comes with several notable drawbacks. It struggles with handling conditional branches, such as those necessary for upwinding techniques or variable switching, due to its limited adaptability to dynamic changes in the model. Additionally, estimating truncation errors, which arise from using a finite number of terms from an infinite series, is difficult, particularly for complex functions, impacting the accuracy of the results. Moreover, the method’s efficiency is constrained by its asymptotic complexity (computational memory requirements), leading to increased computational efforts and resources for larger or more complex models, potentially reducing its effectiveness in such scenarios. The three different linearization techniques are summarized in Table 1.

**Table 1:** Comparison of differentiation methods in reservoir simulation.

Method	Flexibility	Ease of implementation	Error handling	Efficiency	Applicability
Analytical differentiation	Low	Low - Requires extensive and detailed programming	Low - Prone to human error but results in exact Jacobians up to rounding error	High - Efficient computation of Jacobians	Specific to the model; changes require substantial rewrites
Automatic differentiation (AD)	High	Moderate - Automated but can require fine-tuning for specific problems	Low - Accurate up to machine precision	Variable - Depends on implementation and problem	Broad - Applicable across various models with flexibility
Numerical differentiation	High	High - Relatively straightforward to implement	Moderate - Difficulty in bounding truncation error	Low - Limited by asymptotic complexity	Broad - Generally applicable, but less suitable for conditional branches

In general, numerical linearization methods are particularly useful when dealing with small perturbations in fluid flow. When the magnitude of perturbation is small compared to the average value of physical quantities, the linearization methods can provide accurate approximations of the system’s behavior and facilitate the computation of the Jacobian for reservoir simulation. In this paper, we focus on analyzing the impact of linearizing the governing equations using different numerical differentiation methods (finite forward difference (FDF)^23^, finite centered difference (FDC)^23^, and operator-based linearization (OBL)^24^) on the accuracy and efficiency of the physical solution of the flow simulation of a typical deadoil fluid model. Our focus centers on assessing these methods’ effectiveness in adapting to time-dependent variations in reservoir conditions, such as fluid movement, compositional changes, and pressure dynamics. The study aims to quantify the computational efficiency, implementation simplicity, and resource utilization of each method. A significant component of our analysis involves a detailed error assessment, particularly on the accuracy of water saturation predictions, and understanding the implications of these errors on the simulation outcomes. The ultimate objective is to identify the method that offers the best balance of computational efficiency and accuracy in modeling varying reservoir conditions.

## Modelling approach

In this section, we present the modelling framework by describing the governing equations and the different linearization methods implemented in our simulations.

### Governing equations

We assume there are oil, gas, and water components in three phases underground. The governing equations for multi-phase fluid flow in a porous medium are described by the conversation of mass, and Darcy law. The continuity equation is written as;

1 $$\begin{aligned} \frac{\partial }{\partial t} (\phi \rho _\alpha S_\alpha ) + \nabla \cdot (\rho _\alpha \textbf{u}_\alpha ) = \rho _\alpha q_\alpha , \quad \alpha = w, o, g \end{aligned}$$

Where $\phi$ is the porosity of porous medium, $\rho _\alpha$ represents a density of phase, $S_\alpha$ is the saturation of phase,$\textbf{u}_\alpha$ describes Darcy’s velocity and $q_\alpha$ illustrate mass flow rate. Darcy velocity is given below for each phase as;

2 $$\begin{aligned} u_{\alpha } = -k \frac{k_{r\alpha }}{\mu _{\alpha }} (\nabla p_{\alpha } - \rho _{\alpha } g \nabla D) \end{aligned}$$

For a dead oil model, the phases present in the reservoir do not include any gaseous components, resulting in a reduction in mass balance equations:

Water component equation

3 $$\begin{aligned} \frac{\partial }{\partial t} (\phi \rho _w S_w) + \nabla \cdot (\rho _w u_w) = \rho _w q_w \end{aligned}$$

Oil component equation

4 $$\begin{aligned} \frac{\partial }{\partial t} \phi (\rho _o S_o) + \nabla \cdot (\rho _o u_o) = \rho _o q_o \end{aligned}$$

The saturation constraint is used to close the system:

5 $$\begin{aligned} S_o+S_w=1 \end{aligned}$$

### Linearization schemes

Linearization techniques play a crucial role in reservoir simulation by transforming complex nonlinear problems into more manageable linear forms. In what follows, we focus on briefly demonstrating the implementation of different differentiation methods (analytical method, FDF^23^, FDC^23^, and OBL^24^) in our simulation procedure.

#### Finite forward difference scheme

The FDC method is a numerical technique primarily used for solving nonlinear equations. This method approximates the derivative of a differential equation by substituting it with a difference quotient, where a specific step size is chosen to maintain an expected order of truncation error. The formulation of the FDF approximation is algebraic and relies on the value of a dependent variable at a certain solution point, connecting it to the values at adjacent forward points.

The FDF approximation is mathematically represented as:

6 $$\begin{aligned} f'(x) = \frac{f(x+h) - f(x)}{h} \end{aligned}$$

In this equation, $f'(x)$ signifies the estimated derivative of the function $f$ at the point $x$, $f(x)$ is the function’s value at $x$, and $h$ is the interval size over which the difference is calculated.

The precision of the FDF method is heavily influenced by the choice of the step size $h$. Rounding errors can become significant and may dominate the approximation, especially as $h$ is reduced. It is important to consider, however, the inherent errors it introduces, particularly in complex scenarios or when computing higher-order derivatives.

#### Finite central difference scheme

The FDC method is a numerical technique used for approximating the derivative of a function *f*(*x*) at a given point *x*. Compared to FDF, it is often more accurate due to its ability to cancel out more terms in the Taylor series expansion, thus reducing truncation error. The central difference scheme for the first-order derivative is given by the formula:

7 $$\begin{aligned} f'(x) = \frac{f(x+h)-f(x-h)}{2h} \end{aligned}$$

This equation represents an approximation of the derivative, where *h* is a small step size. The central scheme considers the function values at points both before ($x-h$) and after ($x+h$) which leads to its increased accuracy over methods that only consider one side (like the FDF method). However, while reducing the step size *h* improves the accuracy of the FDC approximation, it can also introduce numerical instability. This is due to rounding errors and the limitations of finite precision arithmetic inherent in computational systems. If *h* is too small, these errors can significantly affect the results.

#### Analytical scheme

The analytical scheme for derivative approximation is a fundamental approach in mathematical analysis. Unlike numerical methods, the analytical scheme involves deriving an exact expression for the derivative of a function, based on the principles of calculus. This scheme is typically applied in situations where the function $f(x)$ is well-defined and differentiable in the conventional sense. The derivative is computed as:

8 $$\begin{aligned} f'(x) = \lim _{h \rightarrow 0} \frac{f(x+h) - f(x)}{h} \end{aligned}$$

In this expression, $f'(x)$ represents the exact derivative of the function $f$ at the point $x$, and $h$ approaches zero. This limit definition of the derivative provides an exact value, assuming the function $f$ is continuous and smooth over the interval of interest.

The advantage of the analytical scheme is its precision, as it yields the exact derivative without the approximation errors inherent in numerical methods. However, its applicability is limited to functions that are analytically differentiable and where the limit can be practically computed. In many reservior simulation applications, especially those involving complex nonlinear functions, the analytical scheme may not be feasible, necessitating the use of numerical methods like FDF and FDC.

#### Operator based linearization scheme

Typically, OBL is a technique used to linearize nonlinear equations by representing them as a product of two operators: the current state of the system and the spatial position of the system. OBL is utilized in reservoir simulation to solve the equations governing fluid flow through porous media, and is also applied in various other science and engineering fields, such as heat transfer, computational fluid dynamics, and electromagnetics^24,25^.

In the OBL method, nonlinear partial differential equations are transformed into a linear operator form. This is achieved by constructing the Jacobian as a product of a matrix of derivatives with respect to the state variables. State variables and dependent operators are evaluated at the vertices of the mesh in the parameter space, and a continuous representation of state variables and their derivatives is achieved through multi-linear interpolation in this space. The governing equation using OBL is transformed to the following,

9 $$\begin{aligned} V{\phi {}}_0\left[ {\alpha {}}(\omega {})-{\alpha {}}\left( {\omega {}}_n\right) \right] + \Delta {}t\sum _{l\in {}L(i)}\hspace{0.55542pt}{}\sum _{j=\alpha }^{n_p}\hspace{0.55542pt}{}\left( {\Gamma {}}{\Phi {}}_{\alpha } {\beta {}}_{\alpha }(\omega {}) \right) +\theta {}(\xi {},\omega {},u) = 0 , \end{aligned}$$

where,

- $\omega$ is a state-dependent parameter.
- $\psi$ is a space-dependent parameter.
- $${\alpha {}}(\omega {})=\left( 1+c_r\left( p-p_{ref}\right) \right) \sum _{\alpha }^{n_p}\hspace{0.55542pt}{}{\rho {}}_{\alpha }s_{\alpha }$$
- $${\beta {}}_{\alpha }(\omega {})={\rho {}}_{\alpha }k_{\alpha }/{\mu {}}_{\alpha }$$
- $$\theta {}(\xi {},\omega {},u)=\Delta {}t\sum _{\alpha }^{n_p}\hspace{0.55542pt}{}{\rho {}}_{\alpha }q_{\alpha }(\xi {},\omega {},u)$$

where $\omega$ and $\omega _n$ are nonlinear unknowns on the current and previous timestep, respectively, $\theta (\xi , \omega , u)$ is the source term. *V*, $\phi _0$, and $c_r$ are initial volume, porosity, and rock compressibility, respectively, which represent the reservoir-rock properties, and $\rho _{\alpha }$, $k_{r{\alpha }}$, and $\mu _{\alpha }$ are phase density, phase relative permeability, and phase viscosity, respectively, $\Gamma$ is a constant geometrical part of transmissibility and $\Phi _{\alpha }$ is the phase-potential difference at the interface.

This methodology enables the simplification of complex nonlinear physics and the generic linearization approach’s implementation. Rather than conducting complex evaluations of properties and their derivatives relative to nonlinear unknowns during the simulation, the operators are parameterized in physical space either during the preprocessing stage or adaptively, following the approach outlined in^24,26,27^. The parameter space’s configuration depends on the specific physical problem under investigation. In brief, OBL is often more efficient than other linearization methods, such as those using the Taylor series. However, OBL can be less accurate when dealing with equations that are extremely nonlinear.

### Implementation of the methods

The different linearization schemes are implemented in our in-house multiphase simulator. The simulator is based on a Message Passing Interface (MPI) framework to facilitate parallel computing and has advanced linearization and discretization schemes. It employs an unconditionally stable fully implicit method to guarantee robust solutions^28,29^. An in-depth discussion of the implementation of the OBL technique within an advanced parallel framework for reservoir simulation, using high-order discretization techniques, such as the Mixed Hybrid Finite Element Method (MHFEM) and Mimetic Finite Difference (MFD), can be found in the works of^30–34^,among others, to which we direct the reader. In our computational framework, Newton’s method serves as the nonlinear solver^35,36^, a choice motivated by its robust performance in handling the nonlinearities inherent in the equations governing multi-phase flow in porous media^37^. The method requires an accurate evaluation of the Jacobian matrix as it greatly influences the solver’s accuracy and convergence rate.

For the linear solver, we employ an iterative linear solver with Incomplete LU (Lower-Upper) factorization^38^ with zero fill-in, which is a common preconditioning technique used in iterative solvers for linear systems. This solver is specifically chosen for its effectiveness in dealing with sparse matrices typically encountered in reservoir simulation problems^39–41^. The linear solver operates with a a tolerance level of $10^{-9}$ ensuring high precision in the linear solution phase and, consequently, in the overall simulation results. Using a larger tolerance might expedite the computation but at the risk of compromising the accuracy of the results, particularly in simulations where the precise characterization of fluid behavior is critical. Conversely, a smaller tolerance, while enhancing accuracy, would require more computational resources and increase the simulation time. Our experience indicates that the selected tolerance level of $10^{-9}$ for the ILU solver for dead-oil scenarios optimally supports the Newton method’s convergence, providing a reliable and efficient computational strategy for solving the complex nonlinear systems characteristic of multi-phase flow simulations.

## Applied reservoir simulation cases

In this section, a detailed analysis is presented to assess the accuracy and computational efficiency of the implemented numerical methods for a water flooding reservoir simulation scenario. Our study is structured around two tests:

- One-Dimensional (1D) homogenous test: This test involves water injection into an oil system within a 1D mesh framework. The aim is to compare the simulation results with the water saturation profile derived from the 1D Buckley–Leverett analytical solution. This comparison serves to validate the simulation’s ability to accurately replicate known fluid behaviors in a controlled, simplified setting for the various linearization techniques.
- Three-Dimensional (3D) heterogeneous test: This test employs a 3D mesh based on the SPE10 model, known for its high heterogeneity. In this scenario, water is injected in the center, with four production wells strategically located at the corners. This setup is designed to assess the simulation’s capability to handle complex fluid dynamics in a realistic reservoir environment. The solutions of the numerical methods are matched against the analytical solutions of the governing flow equations for a dead oil model, providing a rigorous comparison of the precision and reliability of the suggested linearization techniques.

Both tests are conducted under two different nonlinear tolerance settings-relaxed and tight-to investigate how the choice of tolerance affects the convergence behavior of the linearization methods and the accuracy of derivative computations. With a relaxed tolerance, we examine the methods’ performance without forcing them to achieve the most accurate physical solution, thereby assessing the derivative accuracy under less stringent convergence criteria. Conversely, a tight tolerance setting ensures that the nonlinear solver converges to a physical solution albeit with varying iteration counts and total computation times, allowing us to compare the efficiency of each implementation.

### Test 1: verification of water flooding with Buckley–Leverett solution

In this test, a simulation of a waterflooding scenario in a 1D mesh is carried out to model the flow of two distinct fluid phases, namely oil and water. The dynamics of this flow are calculated using our simulator’s dead oil nonlinear flow solver, which tracks how the saturation levels of the fluids evolve over time in the direction of flow. We conduct a comparison between the numerical solution obtained using different linearization techniques and the analytical solution for a Buckley–Leverett profile. The performance of the linearization methods is analyzed by examining how different step sizes in the FDC and FDF methods, along with varying resolutions in the OBL method, influence the accuracy and computational efficiency of the simulations across a range of nonlinear tolerances.

#### Linearization accuracy: large nonlinear tolerance of $10^{-1}$

**Figure 1:**
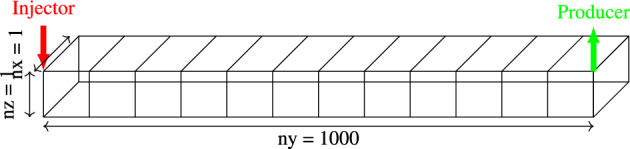
Mesh configuration for waterflooding simulation of Test 1.

**Figure 2:**
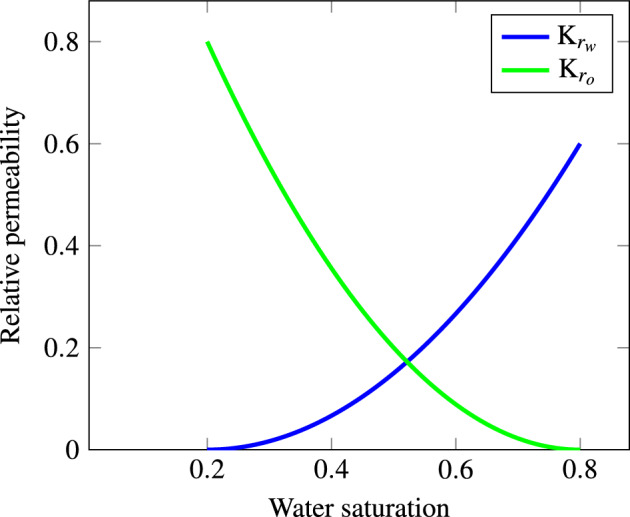
Relative permeability for the water/oil system of Test 1.

For this test, we use a reservoir model with dimensions of $1\times 100\times 1$ m, discretized into a structured grid that consists of 1000 cells aligned along the y-axis, see Fig. 1. On the left side of the mesh, an injection well is located to inject water at a consistent flow rate of 0.159 ${\text{m}}^{3} /{\text{day}}$. At the opposite end, oil is produced from the reservoir, maintained at a steady pressure of 10 bars. The rock and fluid properties are constant throughout the mesh as reported in Table 2 and the behavior of the flow under various saturation conditions is illustrated in the relative permeability curves shown in Fig. 2. The initial distribution of water and oil saturations as well as the operating conditions are tabulated in Table 3.

**Table 2:** Rock and fluid properties of Test 1.

Permeability	Porosity	Pressure	Water density	Oil density	Water viscosity	Oil viscosity
100 md	0.3	230 bar	$1000\hbox { kg/m}^3$	$750\hbox { kg/m}^3$	1 cp	2.5 cp

**Table 3:** Initial and operating conditions of Test 1.

Initial oil saturation	Residual water saturation	Reservoir pressure	Producer pressure	Injector rate
0.8	0.2	230 bar	10 bar	$0.159 m^3$/d

**Figure 3:**
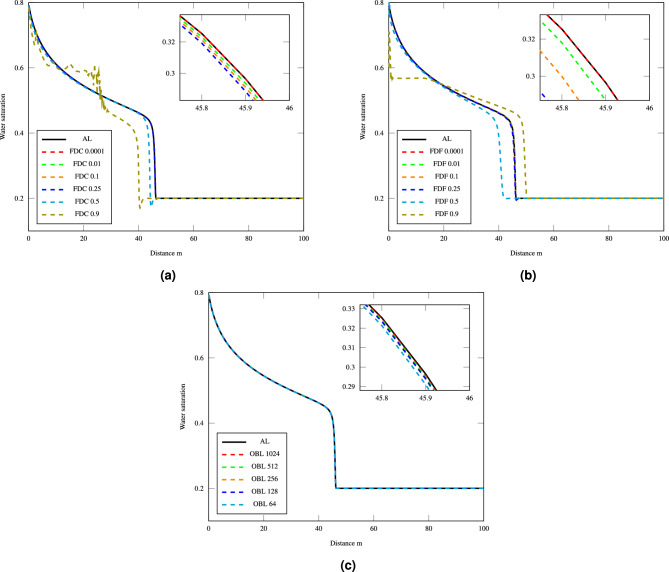
Water saturation profiles for different methods with varying step sizes and resolutions: (**a**) FDC, (**b**) FDF and (**c**) OBL.

The nonlinear tolerance was set to a value of $10^{-1}$ and the resultant water saturation curves using different linearization methods were compared with the analytical solution. The precision of derivative computations within the FDC and FDF methods was fine-tuned by adjusting the step size, *h*, while the resolution of the OBL method was also modified to assess their impacts on the accuracy of water saturation predictions. The water saturation profiles derived using the FDC method are depicted in Fig. 3a, revealing the impact of varying step sizes from very small ($h=0.0001$) to relatively large ($h=0.9$) on the accuracy and stability of the numerical solution. The plot is zoomed in on the shock front area, where the saturation changes most rapidly, to provide a clearer view of how the different step sizes compare to the analytical solution in this critical region noting that finer step sizes like $h=0.0001$ enhance proximity to the analytical solution. Similarly, Fig. 3b illustrates the influence of step size adjustments on the accuracy of the FDF method, where smaller step sizes such as $h=0.0001$ are seen to produce more precise results as illustrated in the zoomed in region. These resuts are consistent as well with the observations of the water saturation profiles in Fig. 3c when various resolutions are employed in the OBL method, ranging from 64 to 1024. For OBL, an increased resolution implies a finer property estimation between the operators point in the method, thus typically yielding a better numerical approximation to the analytical solution. This is evident as the saturation profile for a resolution of 1024 perfectly matches the analytical solution. The comparison of the water saturation profiles across the three methods consistently shows that smaller step sizes and higher resolutions tend to deliver results that closely match the analytical solution and significantly improve the accuracy of the simulation.

**Figure 4:**
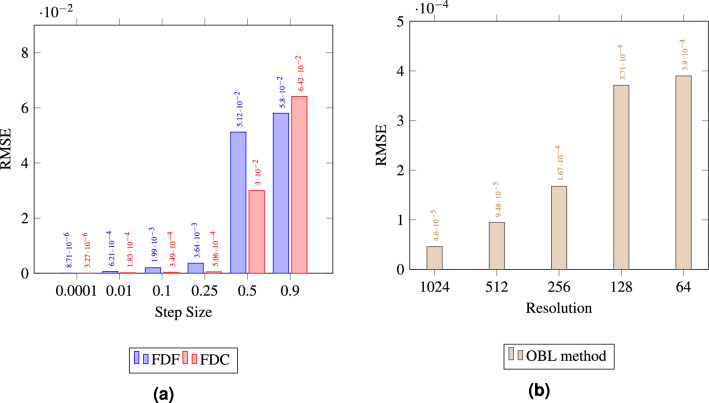
The comparison of RMSE between numerical and analytical solutions for (**a**) FDF and FDC methods, and (**b**) OBL method.

In order to quantify the accuracy of each of the methods, the root mean square error (RMSE) was calculated to compare the quality of the match between the numerical and analytical solutions, as illustrated in Fig. 4a, b. According to the given formula in eq.10, RMSE is a measure of the differences between values predicted by a model and the observed values.

10 $$\begin{aligned} \text {RMSE} = \sqrt{\frac{\sum _{i=1}^{N} (\text {predicted}_i - \text {actual}_i)^2}{N}} \end{aligned}$$

As depicted in Fig. 4a, increasing the step size generally leads to a higher RMSE for both FDC and FDF methods, indicating reduced precision. The FDC method demonstrates better accuracy with a lower RMSE than the FDF method, particularly at a smaller step size of 0.0001. However, at the largest step size of 0.9, the FDF method outperforms the FDC with a lower RMSE, suggesting better precision at this step size. In our case since we are using a high nonlinear tolerance, the simulation is permitted to accept larger errors before considering the solution to have converged, and small differences can lead to significantly divergent outcomes. The FDC method, despite its higher order of accuracy-typically a benefit in systems with minimal nonlinearities-may not necessarily yield superior performance. This potential shortfall is particularly pronounced when the method is applied with a large *h* value in conjunction with a high nonlinear tolerance threshold that fails to capture the small perturbations in the governing equations. Similarly, the OBL method, shown in Fig. 4b, exhibits decreasing RMSE with increasing resolution, confirming that higher resolution correlates with improved accuracy, with the highest resolution of 1024 yielding the most accurate solutions.

#### Linearization efficiency: small nonlinear tolerance of $10^{-8}$

**Figure 5:**
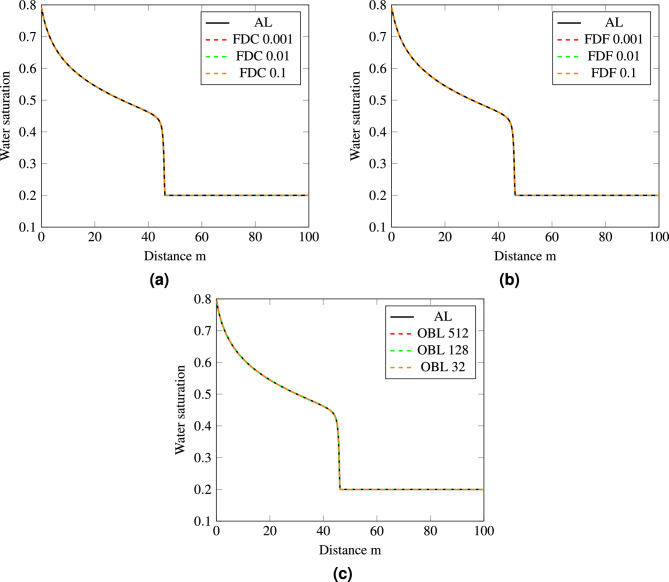
The water saturation profiles for the various methods using a strict nonlinear tolerance of $10^{-8}$: (**a**) FDC, (**b**) FDF and (**c**) OBL.

In this section, we conduct tests on the 1-D flow model using the previously discussed numerical methods (FDC, FDF, and OBL), but with a focus on a smaller nonlinear tolerance of $10^{-8}$. This high-precision tolerance ensures that the simulation results are obtained with greater accuracy. The primary objective of these tests is to examine how the simulation metrics-specifically, the number of iterations and computational time-are affected when all methods are constrained to converge by this strict nonlinear tolerance. The provided plots in Fig. 5 display water saturation profiles derived using FDC, FDF, and OBL with varying step sizes and resolutions. Across all graphs, the saturation profiles closely align with the reference analytical profile, indicating that changes in step size for FDC (0.001, 0.01, 0.1) and FDF (0.001, 0.01, 0.1) methods, as well as changes in resolution for OBL (512, 128, 32), do not significantly affect the accuracy of the results. The reason for this is that all simulations are run with a small nonlinear tolerance, which ensures that the numerical method iterates to a solution that is very close to the exact solution. The question remains as to which particular method is more efficient when simulating this specific 1D flow physical problem.

**Figure 6:**
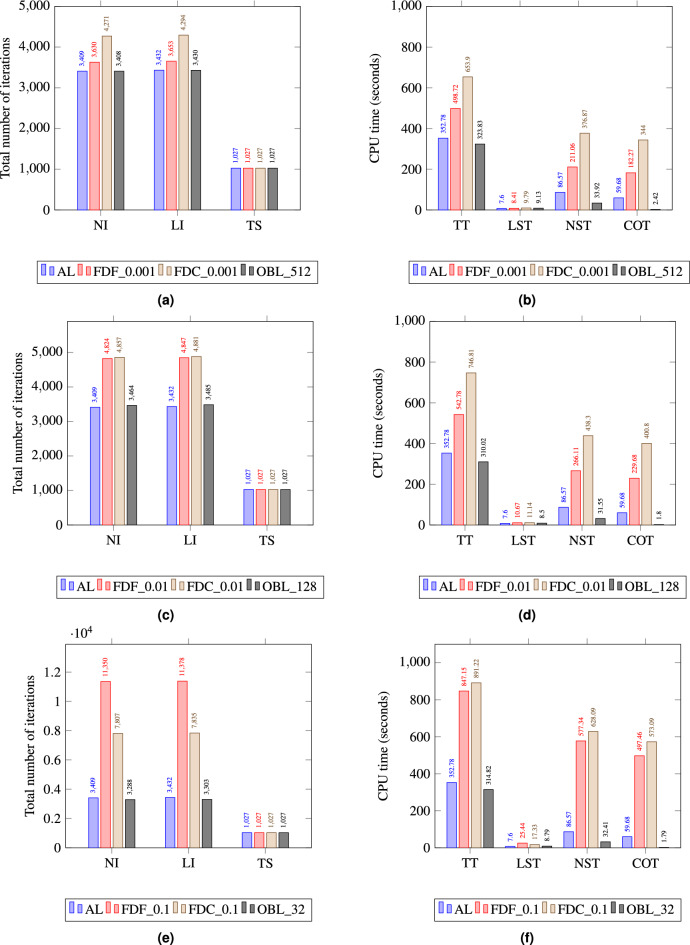
Simulation data of Test 1 for AL,FDF FDC, and OBL schemes. Figure (**a**) and (**b**) depict data at 0.001 stepsize and 512 resolution, Figure (**c**) and (**d**) depict data at 0.01 step size and 128 resolution, and Figure (**e**) and (**f**) depict data at 0.1 stepsize and 32 resolution.

In order to asses each methods’ computational performance, key simulation data highlighting each method’s computing time and convergence performance are plotted in Fig. 6a through 6d for varying step sizes and resolutions. In Fig. 6a for an $h=0.001$ and a resolution of 512, OBL has fewer nonlinear iterations (NI) and linear iterations (LI) compared to both FDF and FDC. This indicates that OBL is more efficient in terms of convergence per iteration at this resolution. All methods have the same time steps (TS) suggesting that the total number of time steps to reach the end of the simulation is predetermined and not affected by the method or its parameters. For the same run, OBL requires significantly less total time (TT) as seen in Fig. 6b, indicating it is the most time-efficient at this resolution. The same colcusions can be extended to the simulation data of Fig. 6c, d for an $h=0.01$ and a resolution of 128 where OBL shows a higher efficiency in terms of the overall performance.

Generally, the nonliner solver (NST) cost takes the biggest cut of the overall CPU time where computing the operators (COT) of the governing equations is the most expensive. This is consistent across the different step sizes and resolutions. However, we notice the that time OBL takes for COT and NST is generally insignificant compared to TT, reflecting a high efficiency of the OBL linearization approach for the simple 1D problem of Test 1. In Fig. 6e, we observe that the NI for FDF is greater than for FDC. Despite the larger number of iterations, the TT for FDF is less than for FDC as shown in Fig. 6f. This might seem counterintuitive at first, but this is attributed to FDF performing more efficient iterations. That is, each iteration takes less time due to simpler calculations or a more straightforward update process in each step. In this specific 1D test, each FDF iteration makes more progress toward the solution despite the higher iteration count. We also notice that at larger step sizes, both the FDF and FDC methods require more iterations to converge comparede to OBL. As *h* increases, the derivative becomes less accurate due to the decreased sampling frequency, which can miss important variations in the function. This can lead to an increased number of iterations being required for the numerical method to converge to an acceptable solution, as it tries to compensate for the loss of detail that comes with a coarser approximation of the nonlinear behavior of the governing equations.

### Test 2: water flooding in a highly heterogeneous reservoir

**Figure 7:**
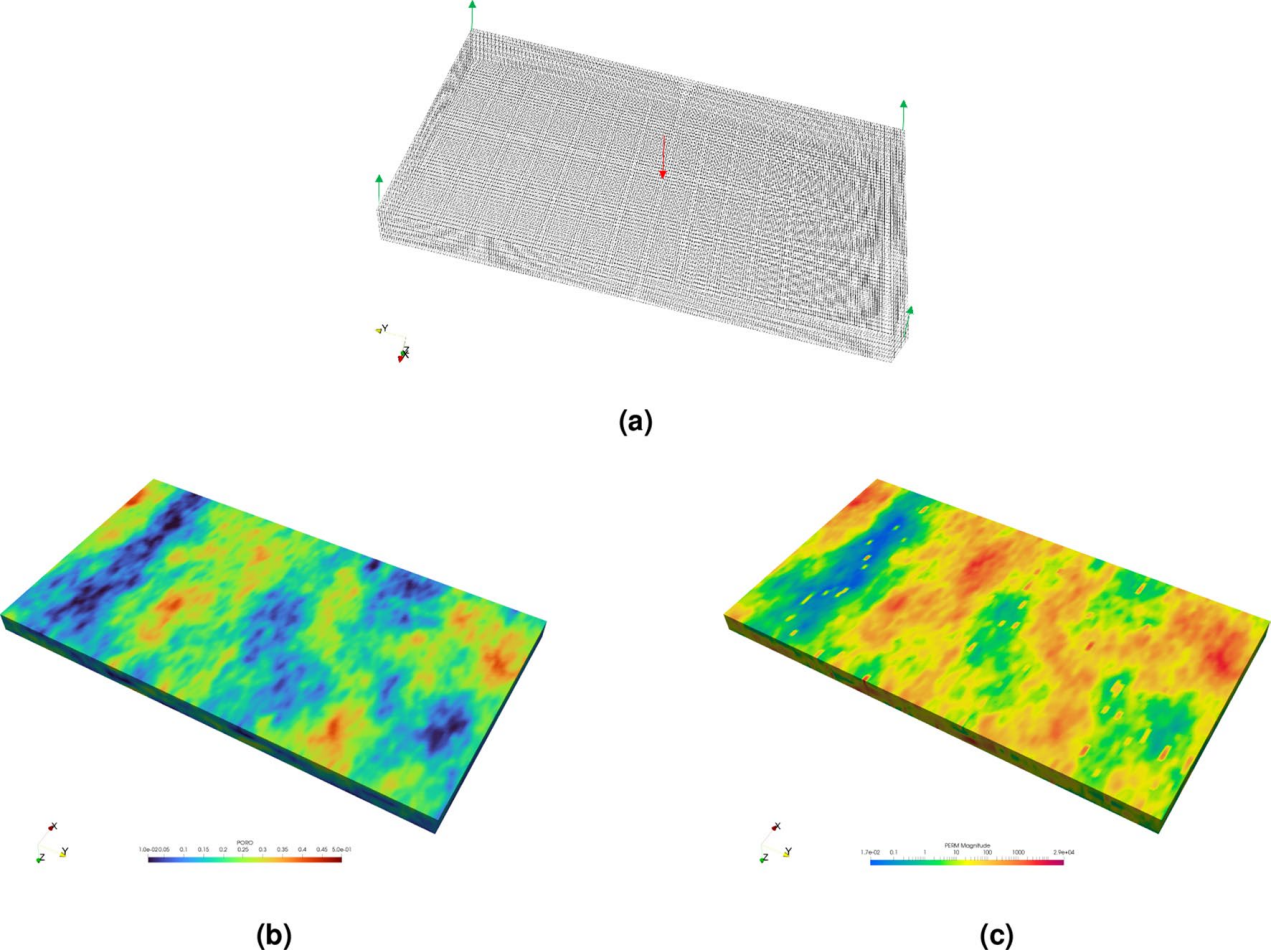
A 3-D mesh representation of the top five layers of the SPE10 model in (**a**) . The producers are shown in green, while the injector is depicted in red. The mesh in (**b**) displays the porosity, while (**c**) illustrates variations in the domain’s permeability.

In this test, we use a 3-D mesh that is based on the top five layers of the SPE10 model as shown in Fig. 7a, where four oil producers are positioned at the corners, and a water injector is placed in the center for a dead oil fluid model. The mesh has dimensions of $240\times 440\times 20$ m, and is characterized by heterogeneous porosity and permeability as shown in Fig. 7b, c. The grid is constructed of 103,880 structured hexaderals, and the parameters used for test two, such as reservoir characteristics, mesh dimensions and fluid properties are provided in Table 4, while the initial and operating conditions are displayed in Table 5 along with the relative permeability curves in Fig. 2 similar to Test 1. This setup is aimed at assessing the numerical linearization techniques’ ability to accurately simulate fluid flow in a complex reservoir model with the aim of comparing the number of iterations and computational time across methods.

**Table 4:** Mesh and fluid properties of Test 2.

Water density	Oil density	Water viscosity	Oil viscosity	Reservoir size	# of cells
$1000\hbox { kg/m}^3$	$750\hbox { kg/m}^3$	1 cp	2 cp	240$\times$440$\times$20 m	103,880

**Table 5:** Initial and operating conditions of Test 2.

Initial oil saturation	Residual water saturation	Reservoir pressure	Producers’ pressure	Injector Pressure
0.8	0.2	300 bar	250 bar	350 bar

#### Linearization accuracy: large nonlinear tolerance of $10^{-1}$

**Figure 8:**
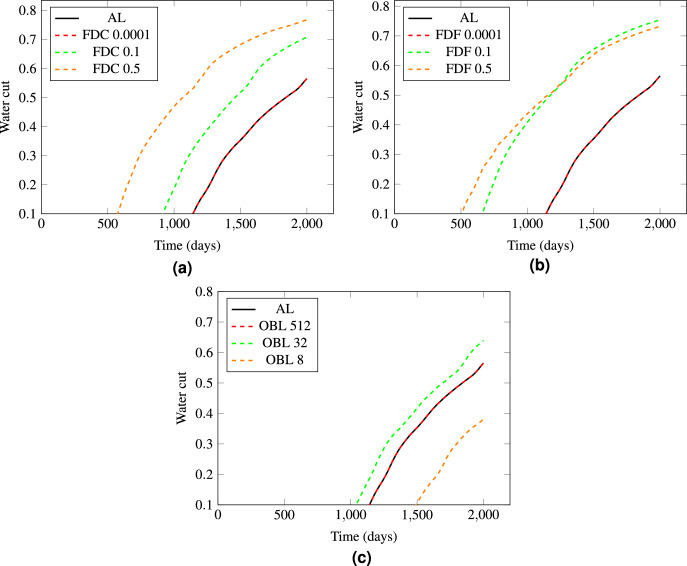
The water cut profiles, depicted in a series of curves from three methods compared to the analytical solution, are shown as follows: Figure (**a**) for FDC, Figure (**b**) for FDF, and Figure (**c**) for OBL.

We performed all the runs using the same linearization techniques of test 1 with a nonlinear tolerance of $10^{-1}$, ensuring that the water cut profiles were taken into consideration. The water cut is the ratio of water produced compared to the total fluids over time. The plots in Fig. 8 showcase three distinct graphs, each representing the progression of water cut over a simulation period of 2000 days for the different linearization techniques . For the FDC method, the smallest step size of $h=0.0001$ resulted in a more accurate water cut profile, as depicted in Fig. 8a, while larger step sizes like 0.1 and 0.5 reduced accuracy massively. The FDF method, shown in Fig. 8b, followed a similar trend in step sizes affecting water breakthrough estimation and accuracy. Only a sufficiently small step size $h=0.0001$ was able to match the analytical solution of the water cut profile. Similarly, the OBL method demonstrated in Fig. 8c showed more precise water cut profiles with higher resolutions such as 512 compared to lower resolutions like 8.

**Figure 9:**
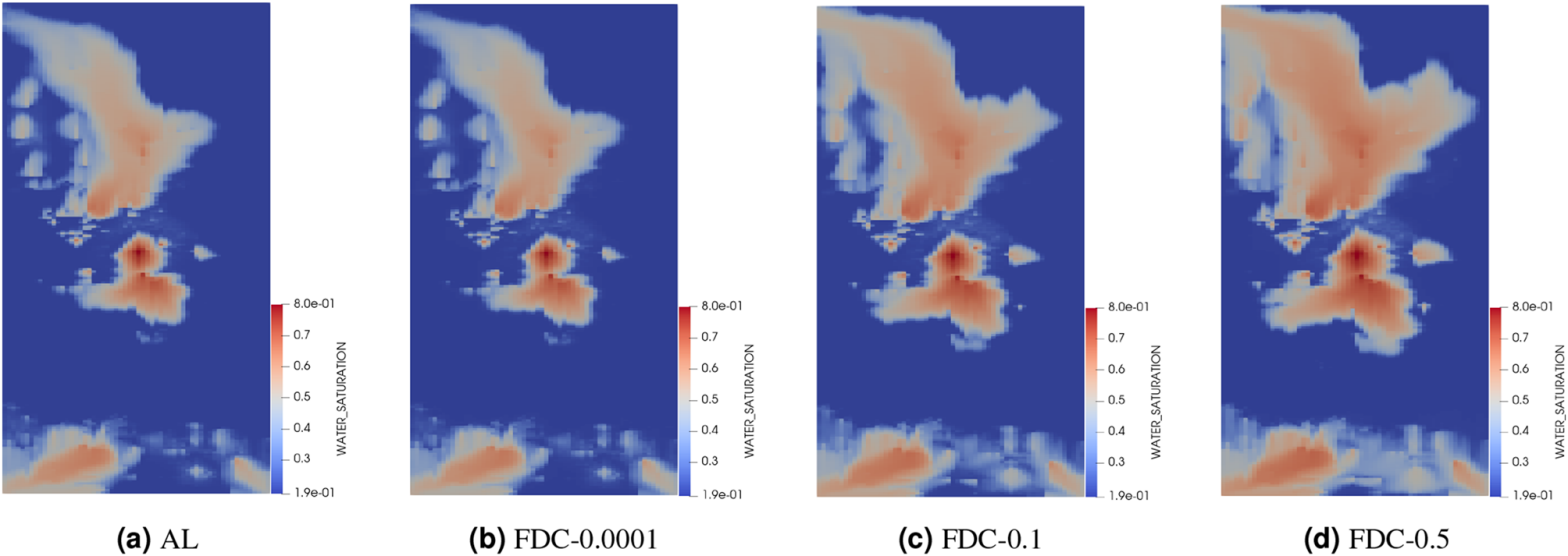
Water saturation maps in the top layer of test 2 after 2000 days showing the analytical solution in (**a**) and the numerical solution using FDC with different step sizes of (**b**) 0.0001, (**c**) 0.1 and (**d**) 0.5.

**Figure 10:**
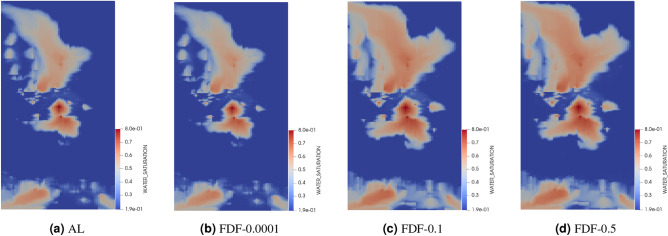
Water saturation maps in the top layer of test 2 after 2000 days showing the analytical solution in (**a**) and the numerical solution using FDF with different step sizes of (**b**) 0.0001, (**c**) 0.1 and (**d**) 0.5.

**Figure 11:**
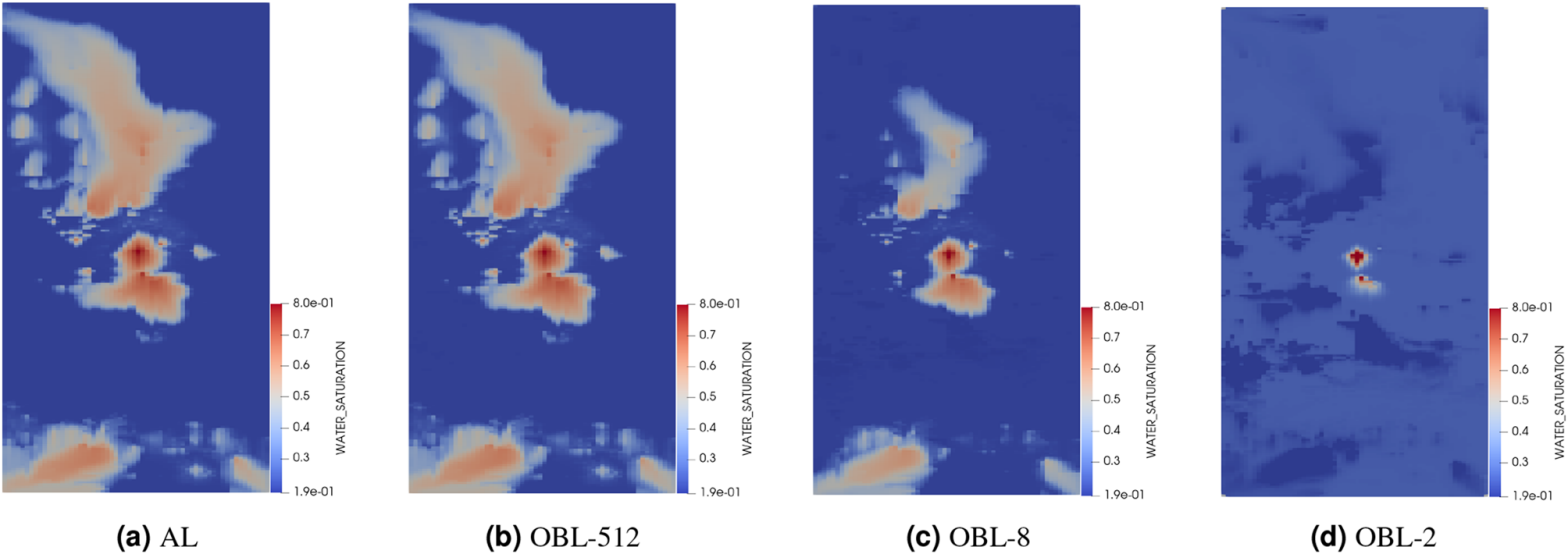
Water saturation maps in the top layer of test 2 after 2000 days showing the analytical solution in (**a**) and the numerical solution using OBL with different resolutions of (**b**) 512, (**c**) 8 and (**d**) 2.

In order to visualize the effect of the step sizes on water saturation distribution in the reservoir, we illustrate the water saturation maps at the four producers over a 2000-day simulation period in Figs. 9, 10 and 11. We aim to compare the accuracy of water breakthrough times across different step sizes for FDC and FDF methods and varying OBL resolutions against the analytical solution. In Fig. 9, as the step size increases from 0.0001 to 0.5, there is a noticeable deviation from the analytical solution, indicating a loss of accuracy in predicting the water breakthrough. The smallest step size (0.0001) closely mirrors the analytical solution, suggesting higher ability in capturing the heterogeneity of the reservoir and hence, more accurate modeling of the water front’s progression. Similarly, the FDF method exhibits a close match to the analytical solution at the smallest step size (0.0001), with larger step sizes like 0.1 and 0.5 leading to less accurate representations of the water breakthrough, as shown in Fig. 10. When the OBL methods is used in Fig. 11, there is a clear trend where higher resolutions better capture the water saturation pattern as depicted in the analytical solution, suggesting a more accurate prediction of water breakthrough times. At low resolutions(OBL-8 and OBL-2), the model’s ability to accurately predict water breakthrough reduces, with the OBL-2 resolution showing significant disparities from the analytical model as the saturation patterns become overly broad and dispersed. The comparison of the water saturation maps across the methods underlines a consistent theme: finer approximation, whether by step size or resolution, is essential for accurate representation of complex physical processes within heterogeneous reservoirs.

**Figure 12:**
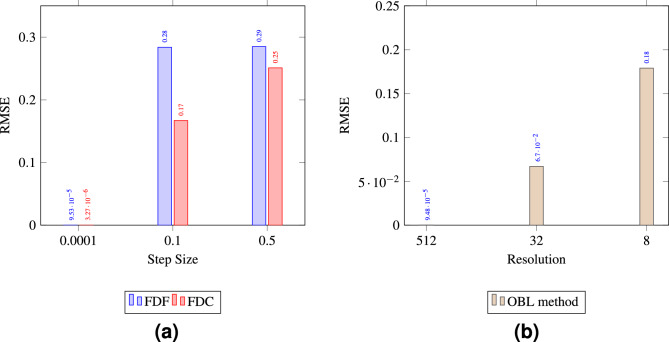
The comparison of RMSE between numerical and analytical water cut profiles of Test 2 for (**a**) FDF and FDC methods, and (**b**) OBL method.

In general, the estimation accuracy of numerical water cut profiles is closely linked to the high heterogeneity of the simulated reservoir. Larger step sizes in FDC and FDF or large resolution in OBL result in derivative estimations over broader areas, smoothing out essential variations in reservoir properties and leading to inaccuracies in the water cut profiles. Conversely, smaller step sizes allow for more detailed representation of the derivative and captures the changes in the physical properties of the flow that are necessary for precise simulation. This conclusion is evident by observing the RMSE between the numerical and analytical water cut profiles plotted in Fig. 12. The data suggests that both smaller step sizes for FDF/FDC and higher resolutions for OBL enhance the accuracy of the numerical solutions in a trend that is similar to the one observed in Test 1. However, this is considered a trade-off, as increased accuracy comes at the cost of higher computational demands which we will explore in the next section.

#### Linearization efficiency: small nonlinear tolerance of $10^{-4}$

**Figure 13:**
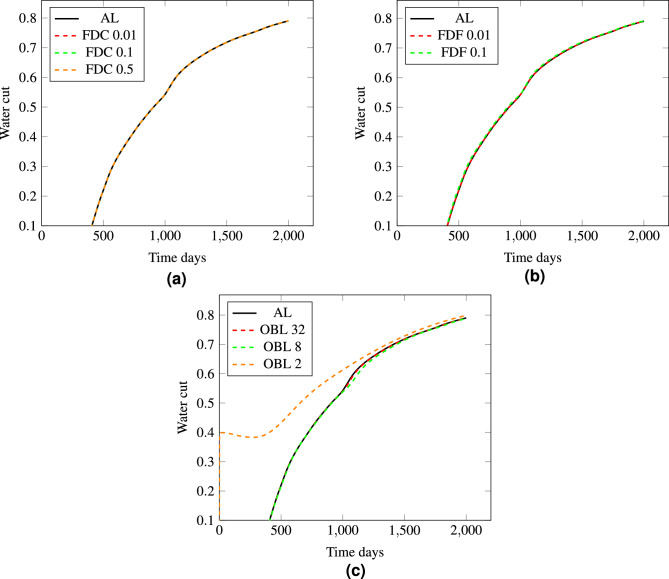
The analysis of the water cut profiles involves comparing three distinct methods with an analytical solution. The results obtained from each method are presented in separate figures. Figure (**a**) illustrates the FDC, Figure (**b**) depicts the FDF and Figure (**c**) displays the OBL.

In these series of runs, we reduced the nonlinear tolerance to $10^{-4}$, which is a smaller tolerance that typically yields a more accurate solution but potentially more computationally intensive simulation. The presented plots in Fig. 13 illustrate water cut over time, comparing the results of the FDC, FDF, and OBL methods to the analytical solution with the reduced nonlinear tolerance. The FDC and FDF methods in Fig.13a, b, with step sizes of 0.01 and 0.1, show a close match to the analytical solution because of the stricter convergence criteria which forces the numerical solution to be more precise. Contrary to the FDC method, the FDF method at step size 0.5 completely failed to converge. This is explained by the fact that when the step size is increased, the discrete representation of the derivative becomes a coarser approximation of the actual gradient in the reservoir. If this approximation is too coarse, the nonlinear solver may not be able to find a solution that falls within the specified tolerance range, leading to a failure to converge. Our strict tolerance demands a high level of precision in the numerical solution, which a large step size for the FDF method does not provide, especially in a highly heterogeneous reservoir where fine-scale variations significantly influence fluid flow behavior.

On the other side, the OBL method exhibits a noticeable difference in performance at varying resolutions. At a resolution of 32 and 8, the water cut profile closely follows the analytical solution, indicating accurate simulation results as seen in Fig. 13c. However, when the resolution is reduced to 2, the OBL method fails to predict the water cut accurately, displaying a significant deviation from the analytical curve. At this coarse resolution, essential details about fluid flow and saturation changes over small spatial scales are lost, leading to an oversimplified model that cannot reflect the complex flow of water and oil in the porous medium.

**Figure 14:**
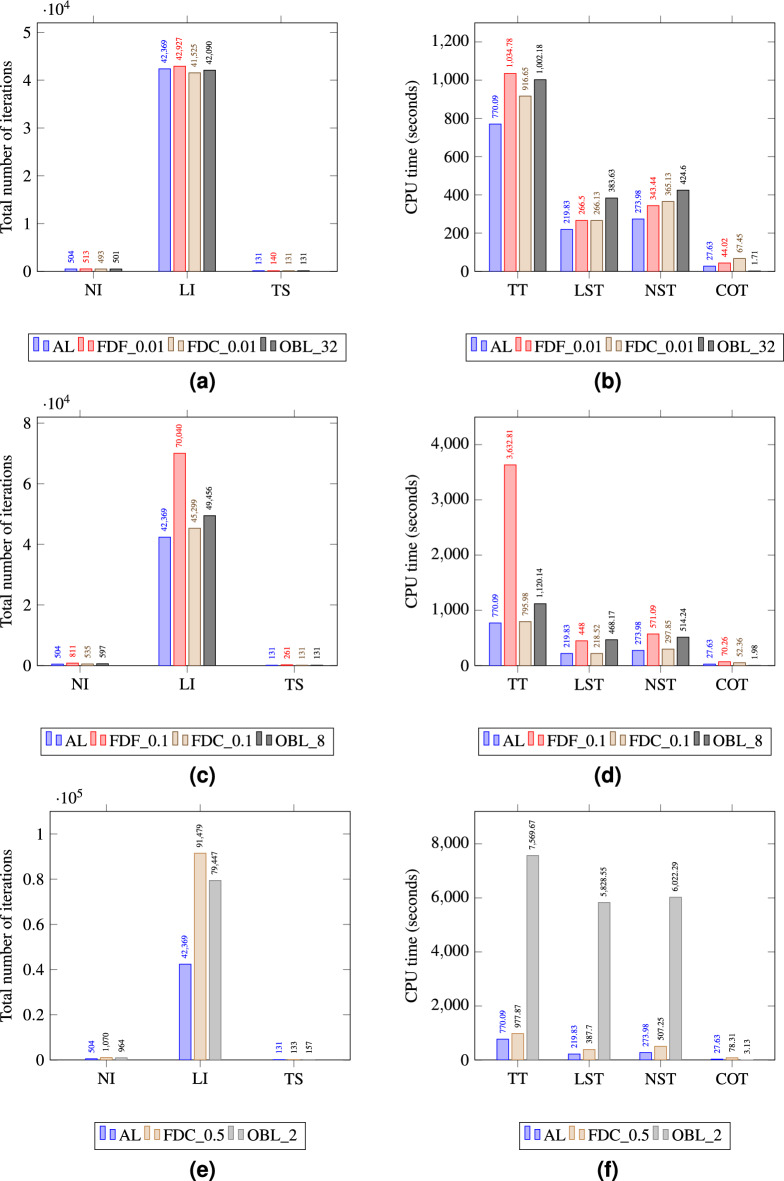
Simulation data of Test 2 for AL,FDF FDC, and OBL schemes. Figure (**a**) and (**b**) depict data at 0.01 stepsize and 32 resolution, Figure (**c**) and (**d**) depict data at 0.01 step size and 8 resolution, and Figure (**e**) and (**f**) depict data at 0.5 stepsize and 2 resolution.

The simulation data regarding computational performance are compared across the four methods and shown in Fig. 14. For the FDF and FDC methods with smaller step sizes (0.01), the number of iterations and computing times in Fig. 14a, b are relatively low for such a complex test, indicating efficient performance. However, as the step size increases to 0.1 for FDF and to 0.5 for FDC, there is a notable increase in computational time, particularly in LST and NST suggesting that larger step sizes lead to more complex and time-consuming numerical problems as a result of coarser derivative estimations. The OBL method at higher resolutions (OBL-32 and OBL-8) shows a relatively low computational time, aligning with efficient simulation performance. Yet, when the resolution is extremely coarse (OBL-2), the method requires significantly longer computational times, specifically in NST and LST, indicating difficulties in solving the linearized system accurately due to the loss of resolution in the physical domain. The analytical solution had generally the lowest simulations time and lowest iteration count in comparison with the the other linearization techniques. Moreover, we notice that the analytical solution performed the best in comparison with the other linearization methods as calculating the exact values of the derivatives of the governing equations produce accurate representation of the flow in such a complex case.

## Overview of main findings

Our study addresses specific challenges in reservoir simulation focusing on the behavior of fluid flow within reservoirs and aims to predict and understand the accuracy and efficiency of different numerical linearization methods used in reservoir simulations. The work centers on two tests, including one test based on 1D flow of a Buckley–Leverett problem and a second test based on a 3D heterogeneous reservoir mesh. The tests specifically examine the challenges posed by complex multidimensional simulations using a 3D heterogeneous mesh where fine-scale variations critically influence the flow behavior. They also consider the impact of changing nonlinear tolerances and step sizes on the accuracy and efficiency of both 1D and 3D flow simulations.

In Test 1, we employed a 1D Buckley–Leverett solution to validate water flooding simulations and noticed that larger nonlinear tolerances resulted in less accurate matching with analytical solutions, thus impacting the simulation accuracy. Conversely, smaller step sizes and larger nonlinear tolerances improved convergence rates for FDF and FDC methods, with the OBL method showing high efficiency at the largest tested resolution of 1024. Test 2, utilizing a 3D heterogeneous mesh based on the SPE10 model, demonstrated that larger step sizes led to deviations from analytical solutions, particularly for FDC and FDF methods, highlighting the need for precise derivative estimations in reservoirs with high heterogeneity.

Generally, analytical solutions are more efficient than numerical solutions when they are available, as they offer exact results without the need for approximation. However, analytical solutions, while exact, can sometimes involve complex calculations that are more computationally intensive than a well-optimized numerical method similar to our case in Test 1 where OBL showed a superior performance across all resolutions. In summary, the following observations can be drawn from the simulation data of Test 1:

- OBL consistently shows lower NI and LI counts across all resolutions, suggesting that it converges with fewer iterations making it more appealing for solving simple problem for its reliability and speed.
- OBL consistently takes less time in TT, LST, NST, and COT across all resolutions, indicating that it is generally more time-efficient than FDF and FDC for this type of problems.
- The TS are consistent across all methods for each case, indicating that the total number of time steps required for the computation does not vary with step size or resolution.
- FDF generally requires more iterations and more time than FDC, suggesting that the latter is more efficient of the two when it comes to varying step sizes.
- FDC generally has the lowest computational efficiency, taking longer to converge, especially as the step size increases.

On the other hand, the simulation data of test 2 indicate a marked efficiency and accuracy of the FDC method in this test compared to the OBL method. This observation is contrasting to the earlier observed performance in test 1 scenario where OBL was more efficient. This shift is explained by the nature of the simulation scenarios and the specifics of each method’s strengths. The FDC’s inherent capability to utilize central points for derivative estimations becomes advantageous in complex simulations, yielding higher accuracy and efficiency when the step size is sufficiently small. In contrast, the OBL method’s efficiency in 1D scenarios does not extend to more complex multidimensional simulations in our tested case where fine-scale variations critically influence the flow behavior. Moreover, we notice that the analytical solution performed the best in comparison with the other linearization methods as calculating the exact values of the derivatives of the governing equations produce accurate representation of the flow in such a complex case.

## Conclusions and future recommendations

The findings from our comprehensive study reveal that each method exhibits distinct strengths and limitations. We conclude that smaller step sizes for FDF and FDC and higher resolutions for OBL yield results that aligned more closely with analytical solutions, thereby improving the accuracy of the simulations. However, this came at the cost of increased computational demand, indicating a trade-off between precision and efficiency. The high heterogeneity of the reservoir required precise derivative estimations, which larger step sizes failed to provide. However, smaller step sizes led to improved accuracy, closely approximating the analytical solutions across all methods. Notably, the FDC method demonstrated superior efficiency and accuracy in heterogeneous media, particularly at larger nonlinear tolerances, while the FDF method and the OBL failed at large step size and low resolutions respectively due to missing important physical features while coarsely linearizing the governing equations. Both tests reveal that smaller step sizes for FDC and FDF and higher resolutions for OBL yield numerical solutions that align closely with the analytical solution, with the OBL method showing the quickest convergence but being less accurate for highly nonlinear equations.

Our future research aims to develop an accurate and fast linearization scheme for complicated physics problems where time-dependency and fluid-dependency play a crucial role on the convergence of the final solution. We suggest several optimization strategies that we will explore in order to enhance the linearization performance:

1. Adaptive Step Size (*h*): Implement a dynamic algorithm that adjusts the step size (*h*) for differentiation based on how each iteration converges. When convergence is slow or unsuccessful, the algorithm should decrease *h* for improved derivative accuracy. This adjustment can be guided by an estimate of the local residual obtained at the nonlinear solver level.
2. Adaptive Method Switching: Begin with a more complex method such as the central difference. If there are problems with convergence, transition to the forward difference method, which, despite having a higher truncation error, can offer greater stability in some scenarios. On the other hand, if the forward difference method is ineffective or converges too slowly, switching back to the central difference may yield a more precise Jacobian.
3. Error-Based Step Size Control based on the Physical Problem: Error-based step size control dynamically adjusts the finite difference step size (*h*) to balance accuracy with computational efficiency. Initially, *h* is chosen based on experience, and after calculating the finite difference derivative, local truncation error is estimated. If the error exceeds the tolerance, indicating that h is too large and leading to inaccurate derivatives, *h* is reduced (for example, $h_{new} = \alpha * h_{current}$), where $\alpha$ is less than 1) to enhance accuracy. Conversely, if the error is significantly below tolerance, h may be increased to speed up computations, though caution is advised to avoid instability due to an overly large step size. The tolerance levels for error assessment are critical and can differ for various derivatives, such as pressure and composition, due to their distinct scales and physical meanings.

There is potential to also combine these strategies in a way to enhance the performance of finite difference methods in the specific context of reservoir simulation, improving both the accuracy of the linearization process and the efficiency of the numerical solver.

## Data Availability

All data generated or analysed during this study are included in this published article.
